# Design, Synthesis and In-Vitro Biological Evaluation of Antofine and Tylophorine Prodrugs as Hypoxia-Targeted Anticancer Agents

**DOI:** 10.3390/molecules26113327

**Published:** 2021-06-01

**Authors:** Ziad Omran, Chris P. Guise, Linwei Chen, Cyril Rauch, Ashraf N. Abdalla, Omeima Abdullah, Ikhlas A. Sindi, Peter M. Fischer, Jeff B. Smaill, Adam V. Patterson, Yuxiu Liu, Qingmin Wang

**Affiliations:** 1 Department of Pharmaceutical Sciences, Pharmacy Department, Batterjee Medical College, Jeddah 21442, Saudi Arabia; 2 Auckland Cancer Society Research Centre, School of Medical Sciences, The University of Auckland, Private Bag 92019, Auckland 1142, New Zealand; chrispguise@hotmail.com (C.P.G.); j.smaill@auckland.ac.nz (J.B.S.); a.patterson@auckland.ac.nz (A.V.P.); 3 State Key Laboratory of Elemento-Organic Chemistry, Research Institute of Elemento-Organic Chemistry, College of Chemistry, Nankai University, Tianjin 300071, China; chenlinwei@mail.nankai.edu.cn (L.C.); liuyuxiu@nankai.edu.cn (Y.L.); wangqm@nankai.edu.cn (Q.W.); 4 School of Veterinary Medicine and Science, University of Nottingham, College Road, Sutton Bonington LE12 5RD, UK; Cyril.Rauch@nottingham.ac.uk; 5 College of Pharmacy, Umm Al-Qura University, Makkah 21955, Saudi Arabia; anabdrabo@uqu.edu.sa (A.N.A.); oaabdullah@uqu.edu.sa (O.A.); 6 Department of Biology, Faculty of Sciences, King Abdulaziz University, Jeddah 21589, Saudi Arabia; easindi@kau.edu.sa; 7 School of Pharmacy, University of Nottingham, Nottingham NG7 2RD, UK; Peter.Fischer@nottingham.ac.uk

**Keywords:** phenanthroindolizidine, antofine, tylophorine, hypoxia, prodrugs, solid tumors

## Abstract

Phenanthroindolizidines, such as antofine and tylophorine, are a family of natural alkaloids isolated from different species of *Asclepiadaceas.* They are characterized by interesting biological activities, such as pronounced cytotoxicity against different human cancerous cell lines, including multidrug-resistant examples. Nonetheless, these derivatives are associated with severe neurotoxicity and loss of in vivo activity due to the highly lipophilic nature of the alkaloids. Here, we describe the development of highly polar prodrugs of antofine and tylophorine as hypoxia-targeted prodrugs. The developed quaternary ammonium salts of phenanthroindolizidines showed high chemical and metabolic stability and are predicted to have no penetration through the blood–brain barrier. The designed prodrugs displayed decreased cytotoxicity when tested under normoxic conditions. However, their cytotoxic activity considerably increased when tested under hypoxic conditions.

## 1. Introduction

Cancer is a complex disease characterized by the loss of control of cell division. It is the second cause of mortality in the world after cardiovascular diseases. Chemotherapy has shown reasonable success in treating cancer; however, the lack of selectivity of chemotherapy drugs, which causes severe side effects, and the emergence of resistance are two major drawbacks preventing the effective clinical use of chemotherapeutic agents [1,2,3,4].

**Figure 1:**
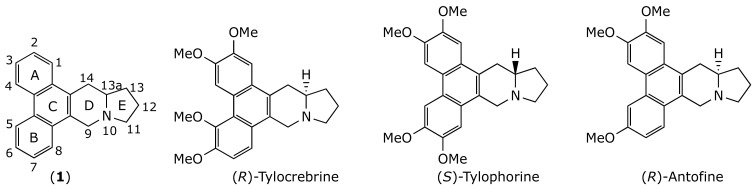
Chemical structure of some phenanthroindolizidine alkaloids.

Natural products have been the major source of the currently available drugs. About 50% of the FDA-approved small molecule anticancer drugs introduced from the early 1940s to the end of 2014 were either natural products or directly derived from them [5]. One example is the phenanthroindolizidine family, a group of alkaloids with general structure (**1**) isolated from different species of *Asclepiadaceas* (Figure 1). These alkaloids display very interesting biological activities, such as pronounced cytotoxicity against different human cancerous cell lines. Prototype compounds, such as tylocrebrine, tylophorine, and antofine (Figure 1), exhibit low nanomolar to picomolar half-maximal growth inhibition (GI_50_) values, as well as effectiveness against multidrug-resistant human cancerous cell lines [6]. Phenanthroindolizidines exert their anticancer activity through a combination of diverse mechanisms [7] such as the inhibition of hypoxia-inducible factor-1 (HIF-1) [8], the inhibition of DNA, RNA and protein synthesis [9,10,11,12], the inhibition of thymidylate synthase [13,14,15], the inhibition of dihydrofolate reductase [13,14,15], the inhibition of Activator Protein-1, and NF-κB, and the down-regulation of cyclin D1 [16,17].

Notwithstanding their therapeutic potential, no compound in this class has yet successfully passed clinical trials. The main drawbacks are their severe central nervous system toxicity and a significant loss of anti-cancer activity when administered in vivo [18,19]. These drawbacks are attributed to the highly lipophilic nature of these alkaloids [6,19] that enables them to cross the blood–brain barrier (BBB) and to undergo high nonspecific protein binding and rapid metabolism. 

One proposed solution has been the use of more polar analogues that cannot cross the BBB and therefore could have less toxic side effects [19]. Nonetheless, efforts to increase the polarity of this type of alkaloid (e.g., by opening the indolizidine ring or introducing hydroxyl groups on the E ring) have led to significant losses of cytotoxicity [20,21]. 

The aim of the present work was to develop polar prodrugs of phenanthroindolizidine alkaloids. The rationale was that the polarity of the new derivatives would prevent them from penetrating the BBB, thereby attenuating their neurotoxicity. The prodrugs were designed to be activated only within the cancerous microenvironment by targeting the unique biochemical alterations in cancer cells, i.e., the low concentrations of molecular oxygen (hypoxia). This, in turn, would further increase the selectivity of the proposed molecules. 

Hypoxia, or oxygen deficiency, is a common feature of most solid tumors and is considered a negative prognostic factor [22]. Hypoxia results from the disorganized tumor vasculature, where the intercapillary distances usually exceed the oxygen diffusion range (around 200 μm) [23]. Hypoxia is responsible, at least in part, for tumor resistance to radio- and chemotherapy [24]. Additionally, hypoxia induces metastasis [25], promotes angiogenesis and vasculogenesis [26,27], and enhances invasiveness [28]. Given the important role of hypoxia in tumor development and progression, and given its relative absence in healthy tissues [29], hypoxia represents an attractive therapeutic target [22,30,31,32]. The main approach to targeting hypoxia consists of the development of bioreductive prodrugs that can be activated in hypoxic tissues by enzymatic reduction [33,34,35,36]. Designing drug delivery systems targeting tumor-hypoxia is another promising approach [37,38,39]. 

**Figure 2:**
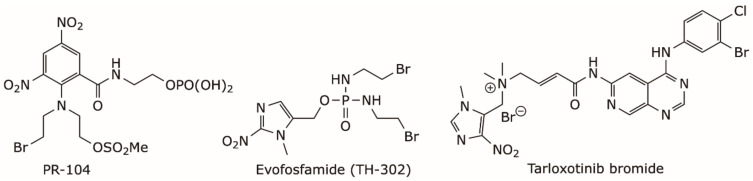
Chemical structure of some hypoxia-targeted nitro(hetero)aromatic-based prodrugs.

Nitro(hetero)cylic derivatives are now well established as prodrugs that can be activated by reductive metabolism mediated by the oxidoreductase enzymes present in human cells [40]. This reduction is repressed by molecular oxygen, making this type of prodrug specific to the hypoxic tumor microenvironment [40]. Some of these prodrugs, such as PR-104 and evofosfamide, also known as TH-302, (Figure 2), have reached Phase II and III clinical trials, either as monotherapy or in combination therapy to treat various types of solid tumors [33,40,41,42]. The permanent positive charge of the tarloxotinib bromide (Figure 2) renders the molecule less permeable to cells than the parent drug, thereby decreasing the toxicity. This attenuation of toxicity permits the administration of such type of quaternary ammonium prodrugs at higher doses than can be achieved with the parent drugs. Additionally, the relative positioning of positive charge and lipophilic head group of this prodrug class leads to sustained tumor residence over time [43]. Tarloxotinib bromide is currently in phase II clinical trials to treat non-small cell lung cancer with HER2 activating mutations [44].

During this work, we successfully applied the approach employed to synthesize tarloxotinib bromide to develop quaternary ammonium salts of antofine and tylophorine alkaloids as hypoxia-targeted prodrugs.

## 2. Results and Discussion

### 2.1. Chemistry

**Figure 3:**
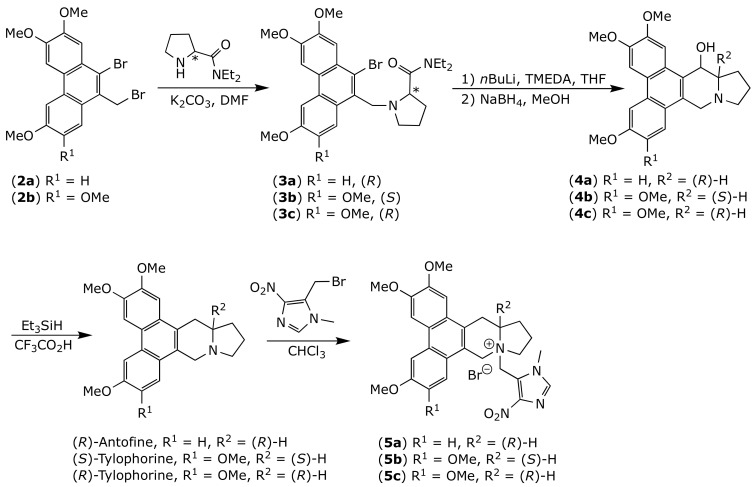
Chemical synthesis of prodrugs **5a**–**c**.

Both enantiomers of tylophorine and (*R*)-antofine were obtained as previously described [45] with slight modification (Figure 3). Briefly, dibromides **2** were alkylated by the corresponding *N*,*N*-diethylpyrrolidine-2-carboxamide. Amides **3** were then treated with *n*BuLi and TMEDA, followed by sodium borohydride, to yield alcohols **4**, which were finally reduced using triethylsilane and trifluoroacetic acid to give the desired alkaloids. The target quaternary ammonium salts **5a**–**c** were successfully synthesized by reacting appropriate precursor alkaloids with 5-(bromomethyl)-1-methyl-4-nitro-1*H*-imidazole [43,46]. The prodrugs **5a**–**c** obtained with reasonable yields (52–68%) after purification by silica gel.

### 2.2. Physicochemical Properties

The physicochemical properties of the new compounds were evaluated (Table 1). The water solubility of the alkaloids was considerably increased, up to 80-fold, when they were transformed to their corresponding quaternary ammonium salts **5a**–**b**. Consequently, these derivatives lost their BBB penetrability, as predicted by the BBB-Parallel Artificial Membrane Permeability Assay (BBB-PAMPA), whereas their parent alkaloids had free passage through the BBB. Additionally, the developed salts had significantly less affinity for plasma proteins than did their parent alkaloids.

**Figure 4:**
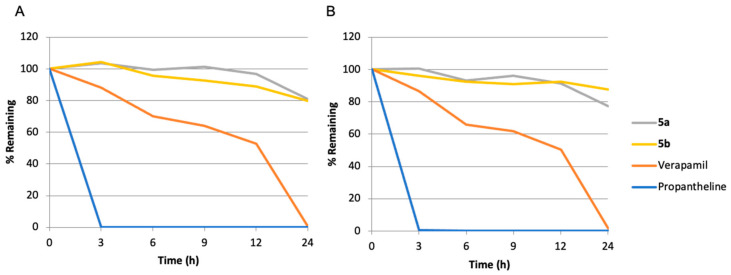
Stability of prodrugs **5a,b** in mouse (**A**), and in human (**B**) plasma.

The chemical and metabolic stability of quaternary ammonium salts was also evaluated to ensure that our prodrugs would not be transformed into the parent alkaloids before arriving at their site of action, the hypoxic tumor. The developed prodrugs displayed high plasma stability when incubated with either mouse or human plasma (Figure 4A,B), attested by t_1/2_ values ranging between 63 and 145 h, Table 2. Interestingly, after 24 h of incubation in either mouse or human plasma, no more than 1% of compounds **5a** or **5b** were transformed into their parent drugs. 

**Figure 5:**
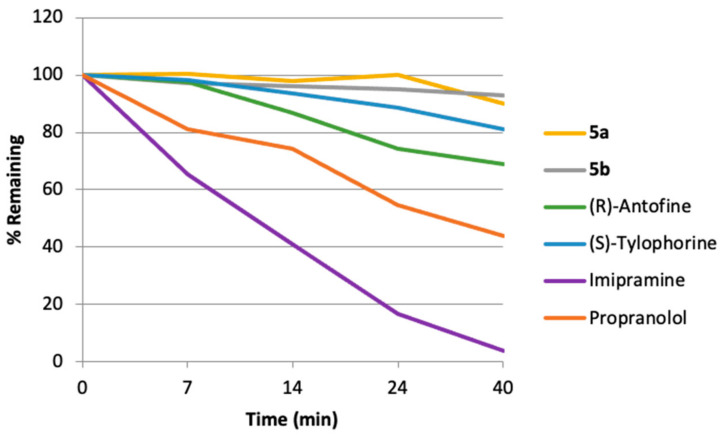
Mouse hepatic microsomal stability for prodrugs **5a,b** and their parent alkaloids.

Similarly, consistent with their reduced lipophilicity the developed quaternary ammonium salts showed significantly higher metabolic stability than was observed for their parent alkaloids following incubation with mouse liver microsomes (Figure 5). The alkaloid half-lives were increased by three- to four-fold upon their transformation to the corresponding ammonium salts (Table 3). It is also noteworthy that less than 1% of compounds **5a** or **5b** was metabolized into the parent drug under these conditions.

### 2.3. Cytotoxic Activity

The cytotoxicity of the compounds **5a**–**c** and their parent alkaloids was evaluated by the 3-[4,5-dimethylthiazole-2-yl]-2,5-diphenyltetrazolium bromide (MTT) assay against 5 cancerous and 2 non-cancerous cell lines (Table 4). All three quaternary ammonium salts **5a**–**c** showed significantly decreased cytotoxicity (up to 1000-fold) against cancerous and noncancerous cell lines alike under normoxic conditions when compared with the parent drugs. 

**Figure 6:**
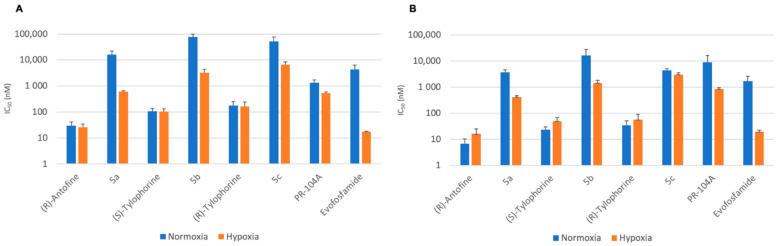
Cytotoxicity of compounds **5a**–**c** and their parent alkaloids under normoxia and hypoxia against (**A**) HCT116 and (**B**) H460 (IC_50_, nM).

We also checked if the designed prodrugs were able to liberate the active alkaloids in hypoxic tumors by testing them under hypoxic conditions using an anaerobic chamber. The three prodrugs **5a**–**c** and their parent alkaloids as well as the reference compounds PR-104A, the parent alcohol form of the phosphate pre-prodrug PR-104, and evofosfamide were tested using the HCT116 and H460 cell lines under both normoxic and hypoxic conditions (Table 5). The prodrugs were much less cytotoxic than the parent drugs, in agreement with our observations using the other cell lines described above (Figure 6).

The extent to which a prodrug is deactivated relative to its parent drug under normoxic conditions is defined as the Deactivation Ratio (DR). The DR can be simply calculated as DR *=* IC_50_ (prodrug)/IC_50_ (parent drug). The developed prodrugs **5a**–**c** displayed excellent DRs ranging between 128 and 719. When tested under hypoxic conditions, the parent antofine and tylophorine drugs showed similar cytotoxicities to those seen under normoxic conditions. By contrast, prodrugs **5a**–**c** showed significantly higher cytotoxicity under hypoxia than under normoxia (Table 4). 

The efficacy of prodrug activation under hypoxic conditions can be expressed by the Hypoxia Cytotoxicity Ratio (HCR), which can be calculated for a given prodrug as: HCR = normoxic IC_50_/hypoxic IC_50_. Prodrugs **5a**–**c** displayed interesting HCRs ranging between 1.5 and 26, thereby confirming the selectivity of these new derivatives for hypoxic tumors (Table 5). 

## 3. Materials and Methods

### 3.1. Chemistry

(13a*R*)-2,3,6-trimethoxy-10-((1-methyl-4-nitro-1*H*-imidazol-5-yl)methyl)-10,11,12,13,13a,14-hexahydro-9*H*-dibenzo[f,h]pyrrolo[1,2-b]isoquinolin-10-ium bromide (5a). To a solution of (*R*)-antofine (0.17 g, 0.48 mmol) in CHCl_3_ (30 mL) was added 5-(bromomethyl)-1-methyl-4-nitro-1H-imidazole (0.16 g, 0.73 mmol), and the reaction mixture was heated at reflux for 20 h under an atmosphere of argon. After the mixture was evaporated under vacuum, the residue was purified by column chromatography on silica gel (CH_2_Cl_2_:CH_3_OH = 10:1) to give the quaternary ammonium salt as a yellow solid (yield 52%). Mp:177–179 °C; purity: >99%; ^1^H-NMR (400 MHz, DMSO-*d*_6_) *δ* 8.24 (s, 2H), 8.14 (s, 1H), 7.87 (d, *J* = 9.0 Hz, 1H), 7.53 (s, 1H), 7.33 (d, *J* = 9.0 Hz, 1H), 5.21 (s, 2H), 5.01 (d, *J* = 17.1 Hz, 1H), 4.87–4.70 (m, 2H), 4.12 (s, 3H), 4.08 (s, 3H), 4.06 (s, 3H), 3.90–3.45 (m, 7H), 2.50–2.37 (m, 1H), 2.32–2.20 (m, 2H), 1.98–1.80 (m, 1H); ^13^C-NMR (100 MHz, DMSO-*d*_6_) *δ* 158.0, 149.7, 149.3, 147.4, 139.8 (2C), 130.5, 124.9, 124.5, 123.9, 122.1, 120.7, 118.3, 115.8, 105.1, 104.8, 104.6, 67.9, 62.6, 56.0, 55.6, 51.5, 50.7, 33.3, 26.8, 24.0, 18.5. HRMS (ESI) calcd for C_29_H_33_N_3_O_5_ (M-Br)^+^ 503.2415, found 503.2301. The spectra are joined in the Supplementary Materials.

(13a*S*)-2,3,6,7-tetramethoxy-10-((1-methyl-4-nitro-1*H*-imidazol-5-yl)methyl)-10,11,12,13,13a,14-hexahydro-9*H*-dibenzo[f,h]pyrrolo[1,2-b]isoquinolin-10-ium bromide (5b). The synthetic procedure was similar to that of compound 5a. Compound 5b was obtained as yellow solid (0.17 g, 58%). Mp:165–167 °C; purity: >99%;^1^H-NMR (400 MHz, DMSO-*d*_6_) *δ* 8.11 (s, 1H), 8.10 (s, 1H), 8.07 (s, 1H), 7.45 (s, 1H), 7.21 (s, 1H), 5.13 (s, 2H), 4.99 (d, *J* = 17.5 Hz, 1H), 4.76–4.66 (m, 2H), 4.07 (s, 3H), 4.06 (s, 3H), 3.99 (s, 3H), 3.96–3.85 (m, 5H), 3.81–3.69 (m, 2H), 3.69–3.49 (m, 2H), 2.46–2.15 (m, 3H), 1.94–1.80 (m, 1H).^13^C-NMR (100 MHz, DMSO-*d*_6_) *δ* 149.9, 149.5, 149.4, 149.3, 147.9, 140.2, 124.5, 124.2, 124.1, 122.9, 121.2, 105.0, 104.93, 104.85, 104.4, 68.2, 63.1, 56.51, 56.48, 56.4, 56.1, 51.9, 51.0, 31.2, 27.2, 24.5, 18.8. HRMS (ESI) calcd for C_30_H_35_N_3_O_6_ (M-Br)^+^ 533.2520, found 533.2403.

(13a*R*)-2,3,6,7-tetramethoxy-10-((1-methyl-4-nitro-1*H*-imidazol-5-yl)methyl)-10,11,12,13,13a,14-hexahydro-9*H*-dibenzo[f,h]pyrrolo[1,2-b]isoquinolin-10-ium bromide (5c). The synthetic procedure was similar to that of compound 5a. Compound 5c was obtained as yellow solid (yield 55%). Mp:185-187 °C; purity: >99%;^1^H-NMR (400 MHz, DMSO-*d*_6_) *δ* 8.11 (s, 1H), 8.10 (s, 1H), 8.08 (s, 1H), 7.45 (s, 1H), 7.20 (s, 1H), 5.13 (s, 2H), 4.98 (d, *J* = 17.4 Hz, 1H), 4.80–4.62 (m, 2H), 4.07 (s, 6H), 4.00 (s, 3H), 3.96–3.84 (m, 5H), 3.82–3.68 (m, 2H), 3.67–3.42 (m, 2H), 2.46–2.14 (m, 3H), 1.94–1.81 (m, 1H).^13^C-NMR (100 MHz, DMSO-*d*_6_) *δ* 149.9, 149.5, 149.4, 149.3, 147.9, 140.2, 124.5, 124.2, 124.1, 122.9, 121.2, 117.9, 104.92, 104.90, 104.8, 104.4, 68.2, 63.1, 56.50, 56.45, 56.4, 56.0, 51.8, 50.9, 49.1, 27.2, 24.4, 18.8. HRMS (ESI) calcd for C_30_H_35_N_3_O_6_ (M-Br)^+^ 533.2520, found 533.2400.

### 3.2. Physiochemical Properties

Water solubility, logD, and PAMPA-BBB were evaluated using the protocols we previously described [47].

### 3.3. Mouse/Human Plasma Stability Test

Incubations were carried out in 6 aliquots of 70 μL each in duplicates. The test compounds (1 μM, final DMSO concentration 0.5%) were incubated at 37 °C with shaking at 100 rpm. Six time points over 24 h (0, 3, 6, 9, 12 and 24 h) have been analyzed. The reactions were stopped by adding 350 μL of acetonitrile with subsequent plasma proteins sedimentation by centrifuging at 5500 rpm for 5 min. Supernatants were analyzed by LC-MS using Shimadzu VP HPLC system including vacuum degasser, gradient pumps, reverse phase column, column oven and autosampler. The HPLC system was coupled with tandem mass spectrometer API 3000 (PE Sciex, Foster City, CA, USA). Both the positive and negative ion modes of the TurboIonSpray ion source were used. The acquisition and analysis of the data were performed using Analyst 1.5.2 software (PE Sciex, API 3000 mass spectrometer, Foster City, CA, USA). The percentage of the test compounds remaining after incubation in plasma and their half-lives (T_1/2_) were calculated.

### 3.4. Mouse Microsomal Liver Stability Assay

Mouse hepatic microsomes were isolated from pooled, perfused livers of Balb/c male mice according to the standard protocol [48]. The batch of microsomes was tested for quality control using imipramine and propranolol as reference compounds. Microsomal incubations were carried out in 96-well plates in 5 aliquots of 40 μL each. Liver microsomal incubation medium contained PBS (100 mM, pH 7.4), MgCl_2_ (3.3 mM), NADPH (3 mM), glucose- 6-phosphate (5.3 mM), glucose-6-phosphate dehydrogenase (0.67 units/mL) with 0.42 mg of liver microsomal protein per ml. Control incubations were performed by replacing the NADPH-cofactor system with PBS. The additional control incubations in PBS (with 3.3 mM MgCl_2_) without added microsomes were carried out in this study. Test compound (2 μM, final solvent concentration 1.6%) was incubated with microsomes at 37 °C, shaking at 100 rpm. The incubations were performed in duplicates. Five time points over 40 min had been analyzed. The reactions were stopped by adding 12 volumes of 90% acetonitrile-water to incubation aliquots, followed by protein sedimentation by centrifuging at 5500 rpm for 3 min. The supernatants were analyzed using LC-MS as mentioned above.

### 3.5. Cytotoxicity Assay

The eight cell lines HEK293 (Human embryonic kidney 293), CHO-K1 (Chinese hamster ovary K1), MCF-7 cells (Human breast adenocarcinoma), HCT116 (human colorectal carcinoma), RKO (Human rectal carcinoma), SW480 (human colorectal carcinoma), H460 (Human lung cancer), and MRC5 (Normal human foetal lung fibroblast), were obtained from the ATCC. The cell lines were routinely grown in alpha minimum essential medium (α-MEM) containing 5% foetal calf serum (FCS). The appropriate number of cells (HCT116 = 400 cells/well, H460 cells = 600 cells/well) were seeded into 96-well plates under normoxic or hypoxic conditions. Cells were plated in 100 µL α-MEM containing 10% FCS, 10mM D-Glucose and 0.2mM 2′-deoxycytidine. After incubation for 2 h, the compounds were added at the appropriate concentration and incubated for further 4 h. The cells were then washed three times with drug-free α-MEM (containing 5% FCS and pen/strep). The plates were then incubated for 5 days under normoxic conditions. The cells were stained with sulphorhodamine B to measure total cells [49]. The IC_50_ was determined by interpolation as the compound concentration reducing staining to 50% of controls on the same plate [50].

## 4. Conclusions

In conclusion, we successfully developed 1-methyl-4-nitro-1*H*-imidazol-5-yl quaternary ammonium salts of phenanthroindolizidine alkaloids as polar hypoxia-selective prodrugs. These new derivatives showed no predicted BBB penetration. Thus, these novel prodrugs should be devoid of neurotoxicity, the main reason for the failure of their parent alkaloids in clinical trials. The developed polar prodrugs **5a–c** showed high chemical and metabolic stability and displayed decreased cytotoxicity when tested under normoxic conditions. However, their cytotoxic activity considerably increased when tested under hypoxic conditions. These new derivatives represent promising lead compounds that can be considered for further preclinical evaluation.

## Figures and Tables

**Table 1:** Physiochemical properties prodrugs **5a,b** and their parent alkaloids.

Compound	PBS (pH 7.4) Solubility μM Mean ± SE	LogD Mean ± SE	Permeability (Papp) Log [10^−6^ cm/s] Mean ± SE	PPB (% of Bound Compound) Mean ± SE
**5a**	154 ± 0	0.69 ± 0.01	<−7	87.0 ± 15
**5b**	162 ± 3	0.38 ± 0.04	<−7	58.9 ± 0.9
(*R*)-Antofine	27 ± 2	3.68 ± 0.03	−5.6 ± 0.58	98.1 ± 0.15
(*S*)-Tylophorine	2 ± 0	3.14 ± 0.02	−5.4 ± 0.17	93.9 ± 0.7
Ondansetron (Reference)	96 ± 3	-	-	-
Mebendazole (Reference)	-	3.26 ± 0.01	-	-
Chlorpromazine (Reference)	-	-	−5.4 ± 0.13	-
Clozapine (Reference)	-	-	−5.1 ± 0.12	-
Ranitidine (Reference)	-	-	<−7	-
Verapamil (Reference)	-	-	-	89.4 ± 0.4

**Table 2:** Mouse and human plasm stability for prodrugs **5a,b,** t_1/2_: half-life.

Compound	t_1/2_, h (Mouse Plasma)	t_1/2_, h (Human Plasma)
**5a**	65.1 ± 0.7	144.7 ± 4.7
**5b**	73.0 ± 1.7	63.3 ± 0.1
Verapamil (Reference)	13.0 ± 1.0	12.2 ± 0.4
Propantheline (Reference)	<3	<3

**Table 3:** Mouse hepatic microsomal stability for prodrugs **5a,b** and their parent alkaloids. K_el_: elimination rate constant, t_1/2_: half-life.

Compound	K_el_, min^−1^	t_1/2_, min
**5a**	0.010 ± 0	286.9 ± 6.5
**5b**	0.002 ± 0	404.0 ± 3.2
(*R*)-Antofine	0.002 ± 0	67.5 ± 2.1
(*S*)-Tylophorine	0.005 ± 0	127.5 ± 3.0
Imipramine (Reference)	0.083 ± 0	8.3 ± 0.4
Propranolol (Reference)	0.021 ± 0	33.5 ± 1.0

**Table 4:** Cytotoxicity of compounds **5a**–**c** and their parent alkaloids under normoxia (nM, IC_50_).

Compound	HEK293	CHO-K1	MCF7	HCT116	RKO	SW480	MRC5
**5a**	5080 ± 519	1680 ± 542	1530 ± 144	1038 ± 21	2034 ± 267	1350 ± 0.126	327 ± 147
**5b**	950 ± 174	3490 ± 843	1498 ± 128	3304 ± 1562	4056 ± 181	7245 ± 490	3313 ± 163
**5c**	-	-	8050 ± 0343	9019 ± 1128	8821 ± 3081	9862 ± 1693	3943 ± 127
(*R*)-Antofine	30 ± 43	33 ± 50	2 ± 0	2 ± 0	6 ± 1	9 ± 02	6 ± 2
(*S*)-Tylophorine	16 ± 26	35 ± 42	62 ± 13	85 ± 6	202 ± 3	69 ± 20	27 ± 2
(*R*)-Tylophorine	-	-	101 ± 88	440 ± 36	338 ± 061	1148 ± 0244	95 ± 6

**Table 5:** Cytotoxicity of compounds **5a**–**c** and their parent alkaloids under normoxia and hypoxia (IC_50_, nM).

	Cell Line	HCT116				H460			
Compound		IC_50_ (Normoxia)	DR	IC_50_ (Hypoxia)	HCR	IC_50_ (Normoxia)	DR	IC_50_ (Hypoxia)	HCR
**5a**		16,471.5 ± 11,571	543	424.7 ± 51	8.8	15,847.0 ± 6339	543	618.0 ± 44	26
(*R*)-Antofine		23.9 ± 7		16.7 ± 9	0.4	29.2 ± 12		25.4 ± 9	0.6
**5b**		4489.0 ± 698	689	1463.3 ± 411	11.3	77,598.3 ± 22,402	719	3220.0 ± 1071	24
(*S*)-Tylophorine		35.1 ± 15		50.1 ± 18	0.5	107.9 ± 28		101.9 ± 32	1.1
**5c**		9055.0 ± 7592	128	3024.7 ± 656	1.5	51,024.7 ± 22,402	293	6500.0 ± 1812	7.8
(*R*)-Tylophorine		1711.0 ± 965		56.4 ± 35	0.6	174.9 ± 78		160.9 ± 77	1.1
PR-104A		16,471.5 ± 11,571	-	860.7 ± 122	10.5	1337 ± 386	-	533.0 ± 47	2.5
Evofosfamide		23.9 ± 7	-	20.1 ± 3	85	4322.7 ± 1931	-	17.2 ± 1	251

## Data Availability

The data presented in this study are available in this article.

## Supplementary Materials

Spectroscopic data (^1^H-NMR, ^13^C-NMR and HRMS) for compounds **5a**–**c** are available online.
