# Supplementary material for: Design, Synthesis and In-Vitro Biological Evaluation of Antofine and Tylophorine Prodrugs as Hypoxia-Targeted Anticancer Agents
Source: Molecules. 2021 Jun 1;26(11):3327. doi: 10.3390/molecules26113327 (PMC8199124; doi:10.3390/molecules26113327)

Design, synthesis and *in-vitro* biological evaluation of antofine and tylophorine prodrugs as hypoxia-targeted anticancer agents

Ziad Omran,[1*] Chris P. Guise,[2] Linwei Chen,[3] Cyril Rauch,[4] Ashraf N. Abdalla,[5] Omeima A. Abdullah,[5] Ikhlas A. Sindi,[6] Peter M. Fischer,[7] Jeff B. Smaill,[2] Adam V. Patterson,[2] Yuxiu Liu,[3] and Qingmin Wang[3]

[1] Pharmacy Department, Batterjee Medical College, 21442 Jeddah, Saudi Arabia

[2] Auckland Cancer Society Research Centre, School of Medical Sciences, The University of Auckland, Private Bag 92019, Auckland 1142, New Zealand

[3] State Key Laboratory of Elemento-Organic Chemistry, College of Chemistry, Collaborative Innovation Center of Chemical Science and Engineering (Tianjin), Nankai University, Tianjin 300071, P. R. China

[4] School of Veterinary Medicine and Science, University of Nottingham, College Road, Sutton Bonington LE12 5RD, UK

[5] College of Pharmacy, Umm Al-Qura University, 21955 Makkah, Saudi Arabia

[6] Department of Biology, Faculty of Sciences King Abdulaziz University, Jeddah 21589, Saudi Arabia

[7] School of Pharmacy, University of Nottingham, Nottingham NG7 2RD, UK

To whom correspondence should be addressed. e-mail: zhomran@uqu.edu.sa

Supplementary materials

Spectroscopic data for compound **5a** Page 2

Spectroscopic data for compound **5b** Page 4

Spectroscopic data for compound **5c** Page 6

Design, synthesis and *in-vitro* biological evaluation of antofine and tylophorine prodrugs as hypoxia-targeted anticancer agents

Ziad Omran,[1*] Chris P. Guise,[2] Linwei Chen,[3] Cyril Rauch,[4] Ashraf N. Abdalla,[5] Omeima A. Abdullah,[5] Ikhlas A. Sindi,[6] Peter M. Fischer,[7] Jeff B. Smaill,[2] Adam V. Patterson,[2] Yuxiu Liu,[3] and Qingmin Wang[3]

[1] Department of Pharmaceutical Sciences, Pharmacy Department, Batterjee Medical College Jeddah 21442, Saudi Arabia
[2] Auckland Cancer Society Research Centre, School of Medical Sciences, The University of Auckland, Private Bag 92019, Auckland 1142, New Zealand; chrispguise@hotmail.com (C.P.G.); j.smaill@auckland.ac.nz (J.B.S.); a.patterson@auckland.ac.nz (A.V.P.)
[3] State Key Laboratory of Elemento-Organic Chemistry, Research Institute of Elemento-Organic Chemistry, College of Chemistry, Nankai University, Tianjin 300071, China; chenlinwei@mail.nankai.edu.cn (L.C.); liuyuxiu@nankai.edu.cn (Y.L.); wangqm@nankai.edu.cn (Q.W.)
[4] School of Veterinary Medicine and Science, University of Nottingham, College Road, Sutton Bonington LE12 5RD, UK; Cyril.Rauch@nottingham.ac.uk
[5] College of Pharmacy, Umm Al-Qura University, Makkah 21955, Saudi Arabia; anabdrabo@uqu.edu.sa (A.N.A.); oaabdullah@uqu.edu.sa (O.A.)
[6] Department of Biology, Faculty of Sciences King Abdulaziz University, Jeddah 21589, Saudi Arabia; easindi@kau.edu.sa
[7] School of Pharmacy, University of Nottingham, Nottingham NG7 2RD, UK; Peter.Fischer@nottingham.ac.uk
* Correspondence: ziad.omran@bmc.edu.sa or ziadomran@hotmail.com

Supplementary materials

Spectroscopic data for compound **5a** Page 2
Spectroscopic data for compound **5b** Page 4
Spectroscopic data for compound **5c** Page 6

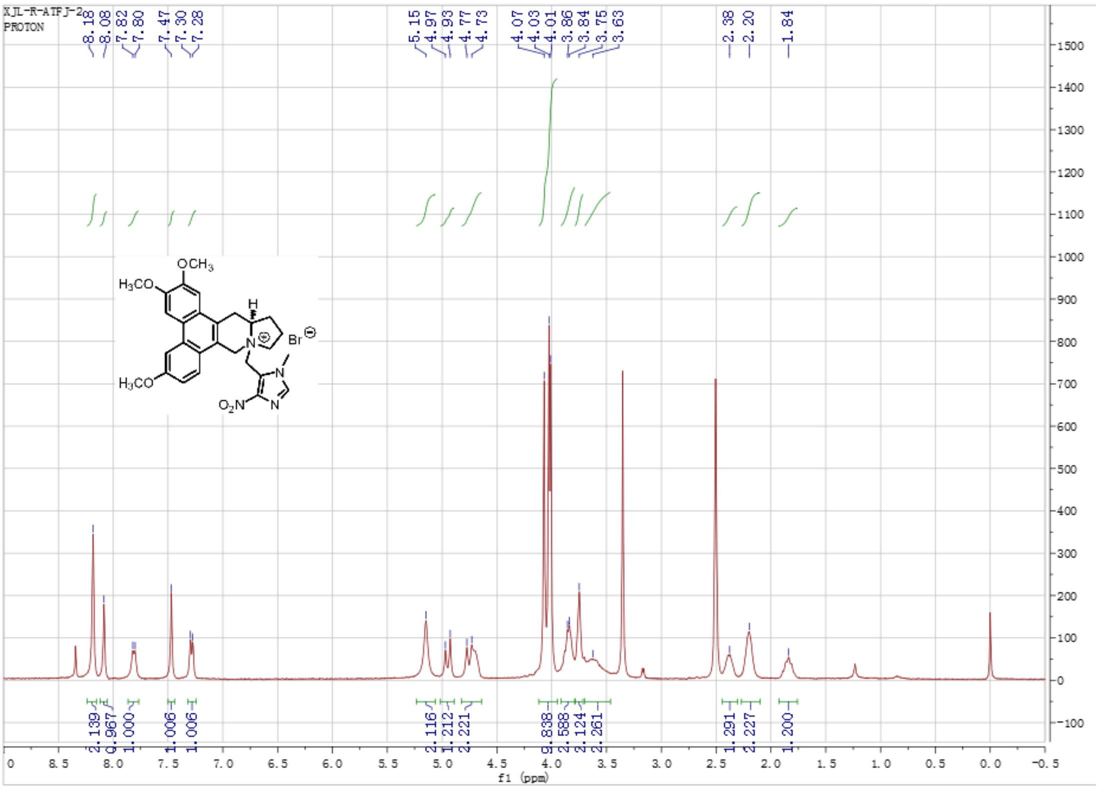

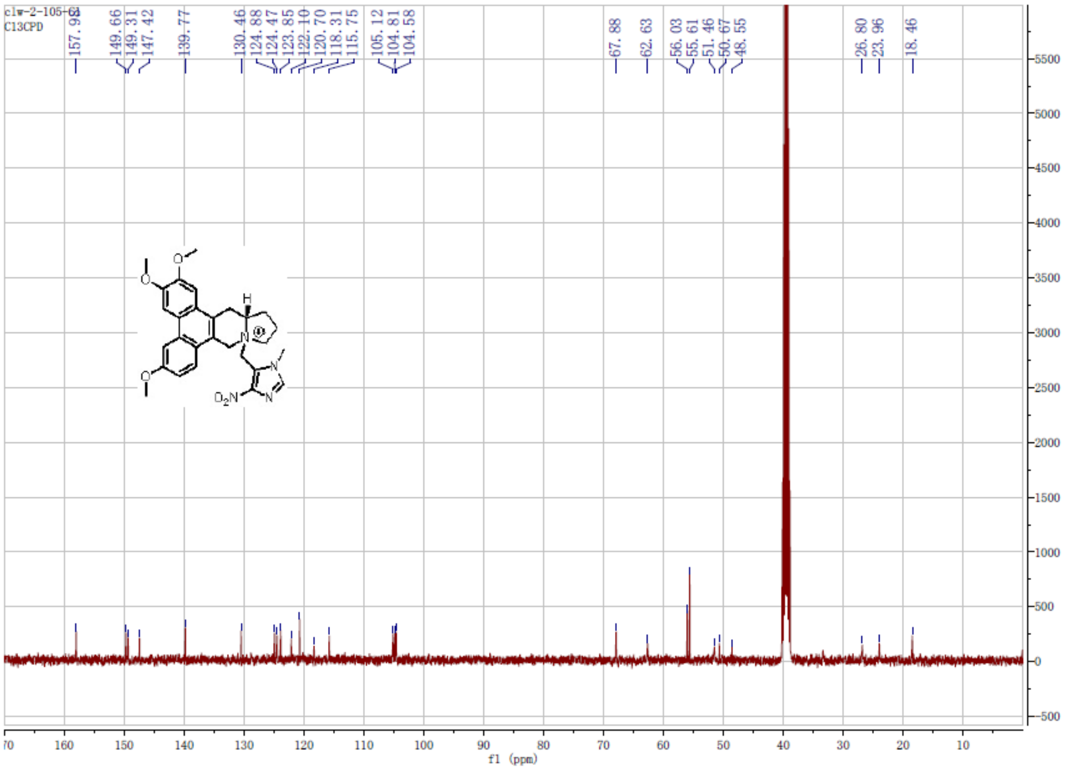

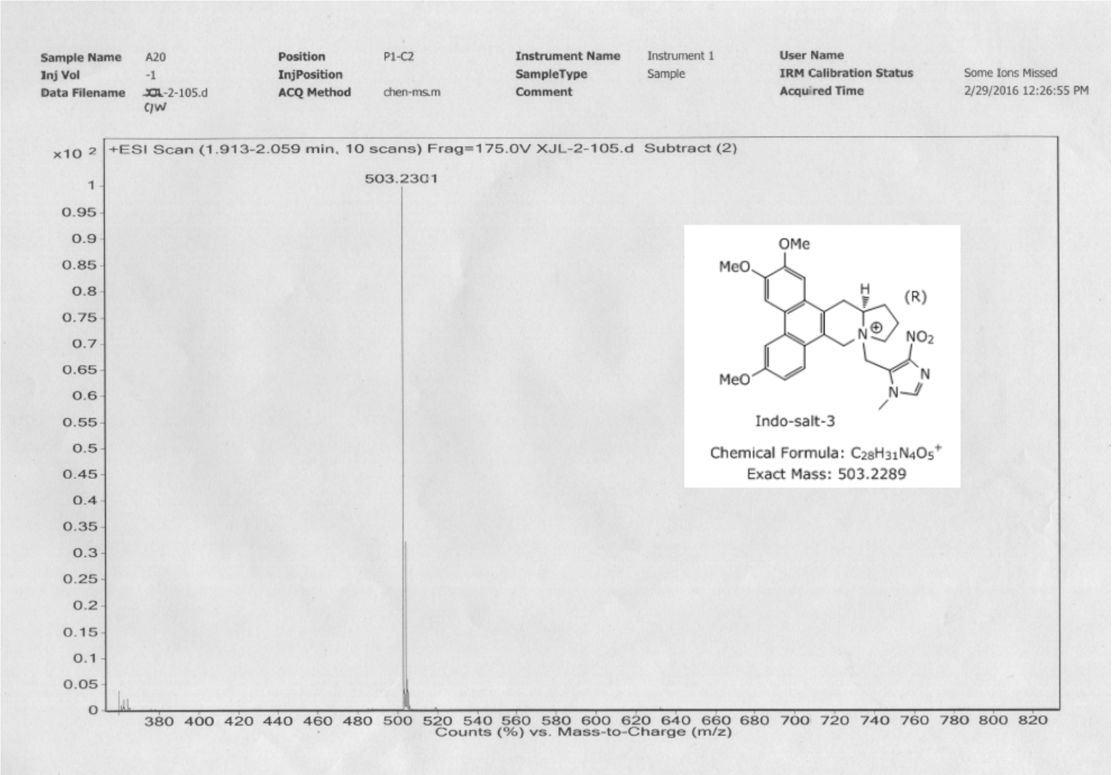
Sample Name A20
Inj Vol -1
Data Filename XJL-2-105.d
CJW
Position P1-C2
InjPosition
ACQ Method chen-ms.m
Instrument Name Instrument 1
SampleType Sample
Comment
User Name
IRM Calibration Status Some Ions Missed
Acquired Time 2/29/2016 12:26:55 PM
x10 2
+ESI Scan (1.913-2.059 min, 10 scans) Frag=175.0V XJL-2-105.d Subtract (2)
503.2301
OMe
MeO
MeO
H
(R)
N
NO2
N
N
Indo-salt-3
Chemical Formula: C28H31N4O5+
Exact Mass: 503.2289
Counts (%) vs. Mass-to-Charge (m/z)

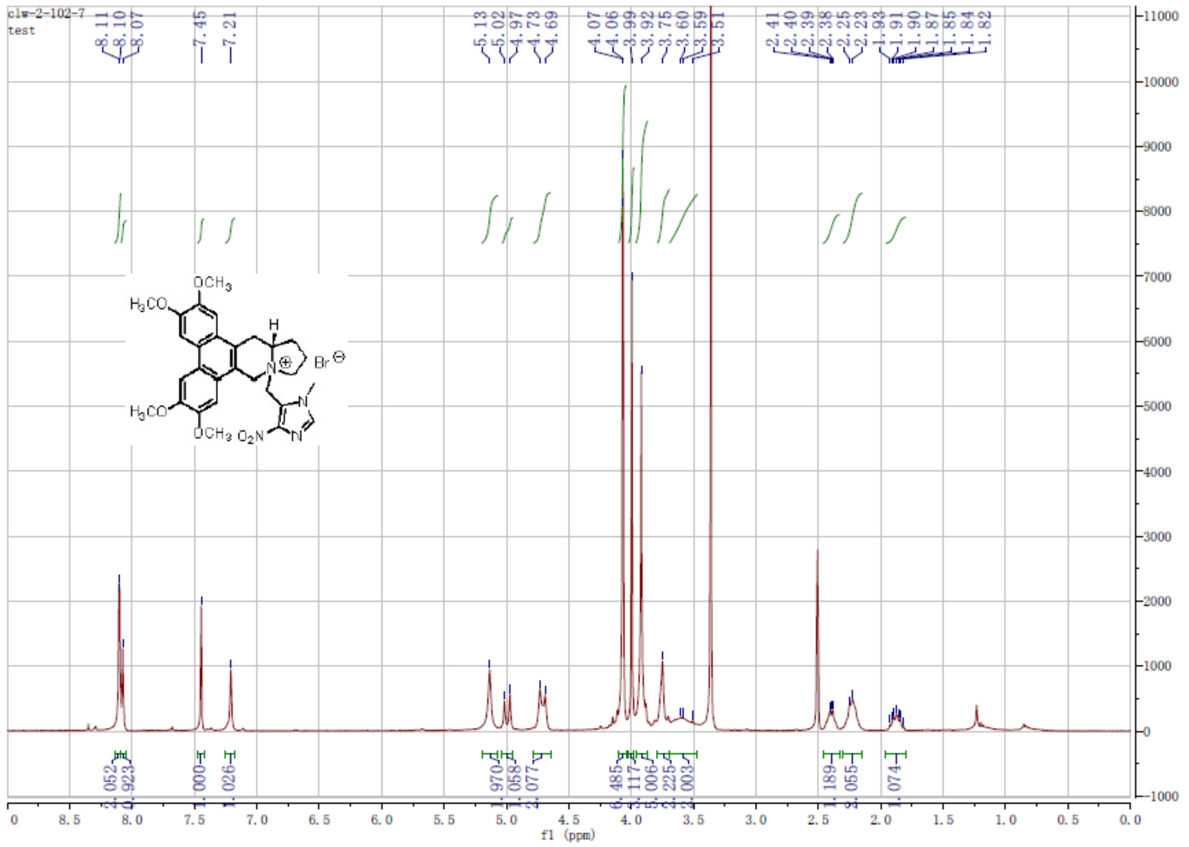
clw-2-102-7
test
8.11
8.10
8.07
7.45
7.21
5.13
5.02
4.97
4.73
4.69
4.07
4.06
3.99
3.92
3.75
3.60
3.59
3.51
2.41
2.40
2.39
2.38
2.25
2.23
1.93
1.91
1.90
1.87
1.85
1.84
1.82
OCH3
H3CO
H
N
Br
H3CO
OCH3
O2N
N
N
f1 (ppm)

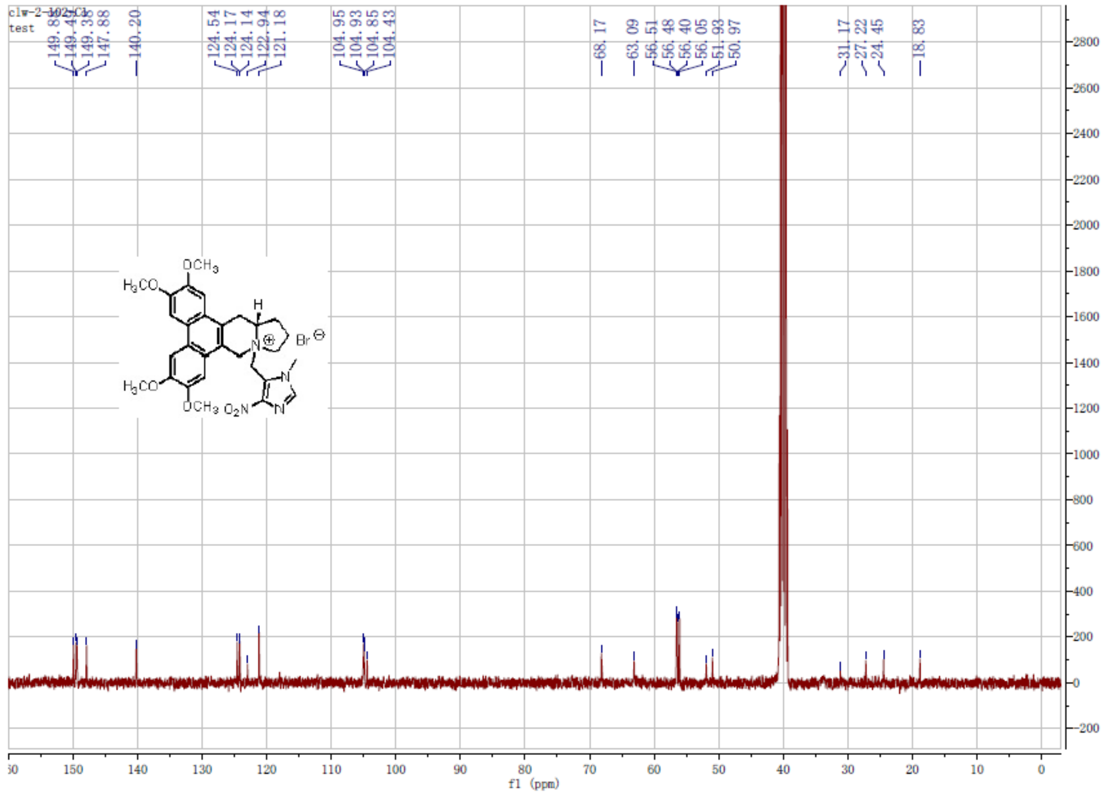
test
147.88
140.20
124.54
124.17
124.14
122.94
121.18
104.95
104.93
104.85
104.43
68.17
63.09
56.51
56.48
56.40
56.05
51.93
50.97
31.17
27.22
24.45
18.83
OCH3
H3CO
H
N
Br
H3CO
OCH3
O2N
N
N
f1 (ppm)

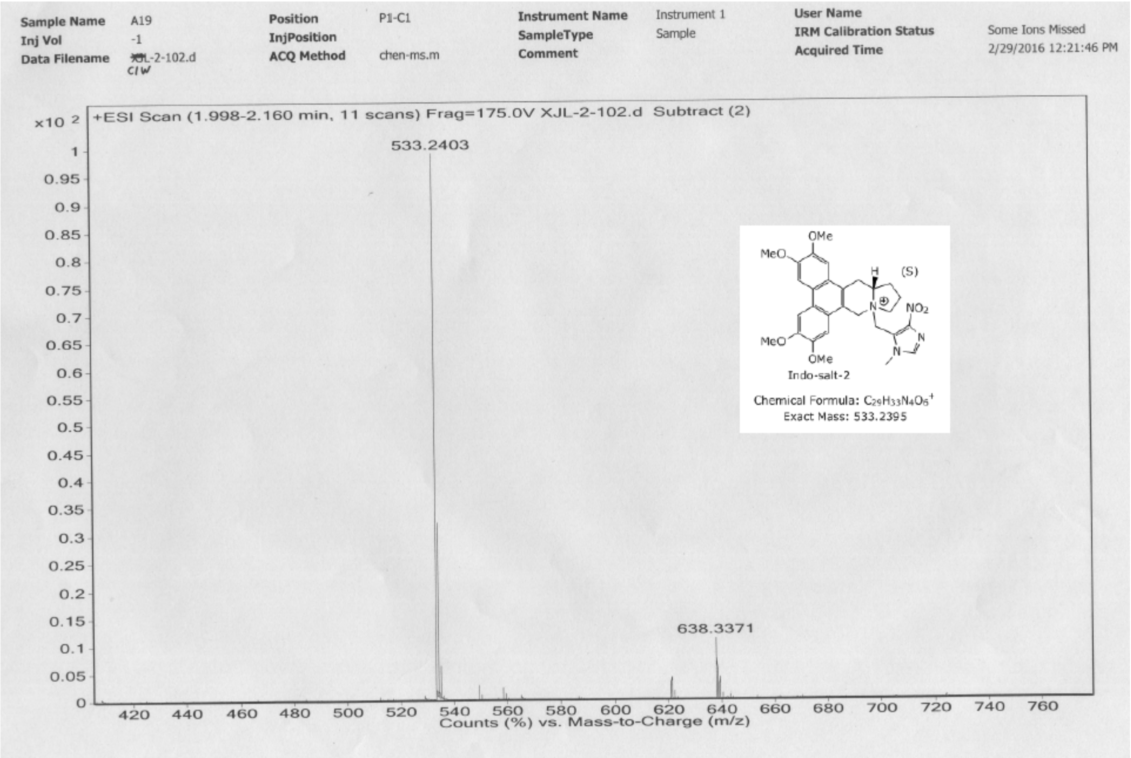
Sample Name A19
Inj Vol -1
Data Filename XJL-2-102.d
Position P1-C1
InjPosition
ACQ Method chen-ms.m
Instrument Name Instrument 1
SampleType Sample
Comment
User Name
IRM Calibration Status Some Ions Missed
Acquired Time 2/29/2016 12:21:46 PM
x10 2
+ESI Scan (1.998-2.160 min, 11 scans) Frag=175.0V XJL-2-102.d Subtract (2)
533.2403
638.3371
Counts (%) vs. Mass-to-Charge (m/z)
OMe
MeO
H
(S)
NO2
MeO
OMe
Indo-salt-2
Chemical Formula: C29H33N4O6+
Exact Mass: 533.2395

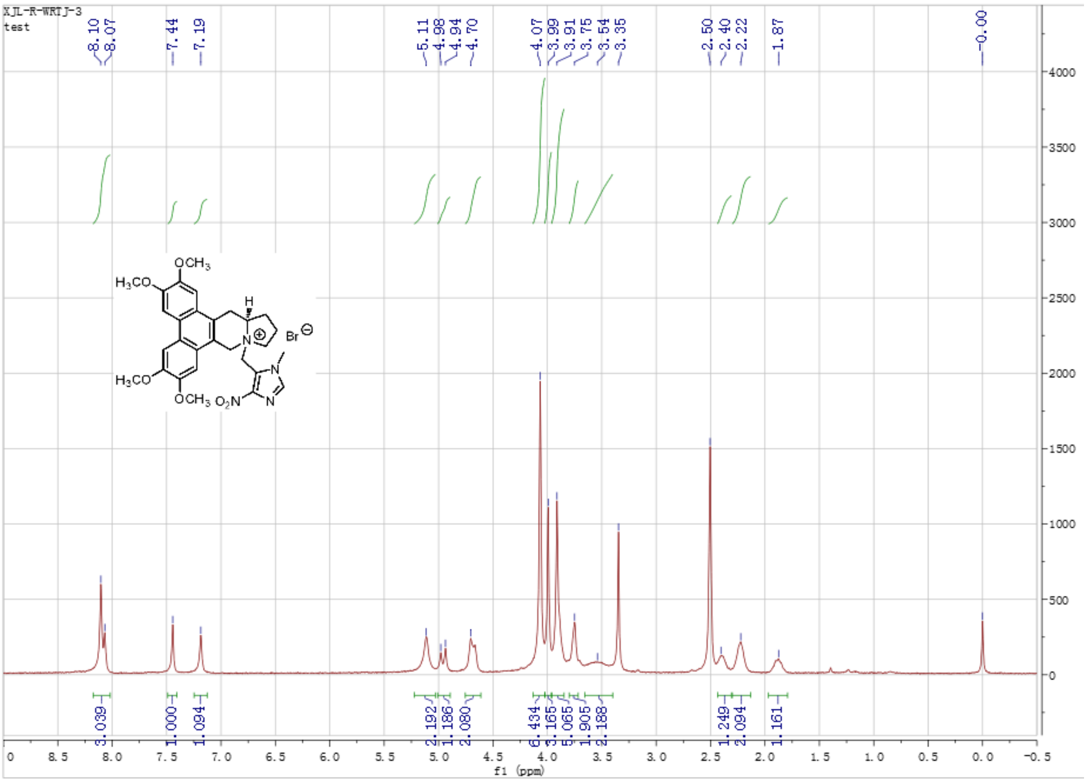
XJL-R-WRTJ-3
test
8.10
8.07
7.44
7.19
5.11
4.98
4.94
4.70
4.07
3.99
3.91
3.75
3.54
3.35
2.50
2.40
2.22
1.87
0.00
OCH3
H3CO
H
N
Br
H3CO
OCH3 O2N
N
N
3.039
1.000
1.094
2.192
1.186
2.080
6.434
3.165
5.065
1.905
2.188
1.249
2.094
1.161
f1 (ppm)

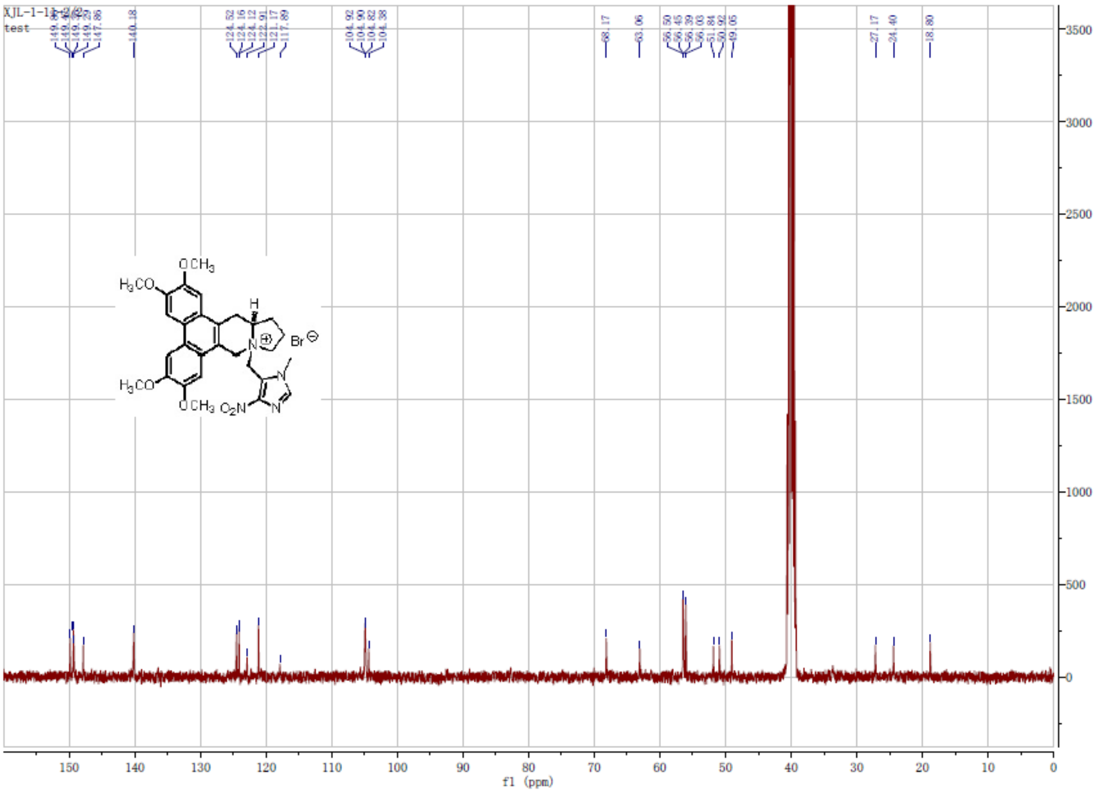
test
140.18
68.17
63.06
27.17
24.40
18.80
OCH3
H3CO
H
N
Br
H3CO
OCH3 O2N
N
N
f1 (ppm)

**Sample Name**	A18	**Position**	P1-B9	**Instrument Name**	Instrument 1	**User Name**	
**Inj Vol**	-1	**InjPosition**		**SampleType**	Sample	**IRM Calibration Status**	Some Ions Missed
**Data Filename**	XJL-1-11.d	**ACQ Method**	chen-ms.m	**Comment**		**Acquired Time**	2/29/2016 12:16:28 PM

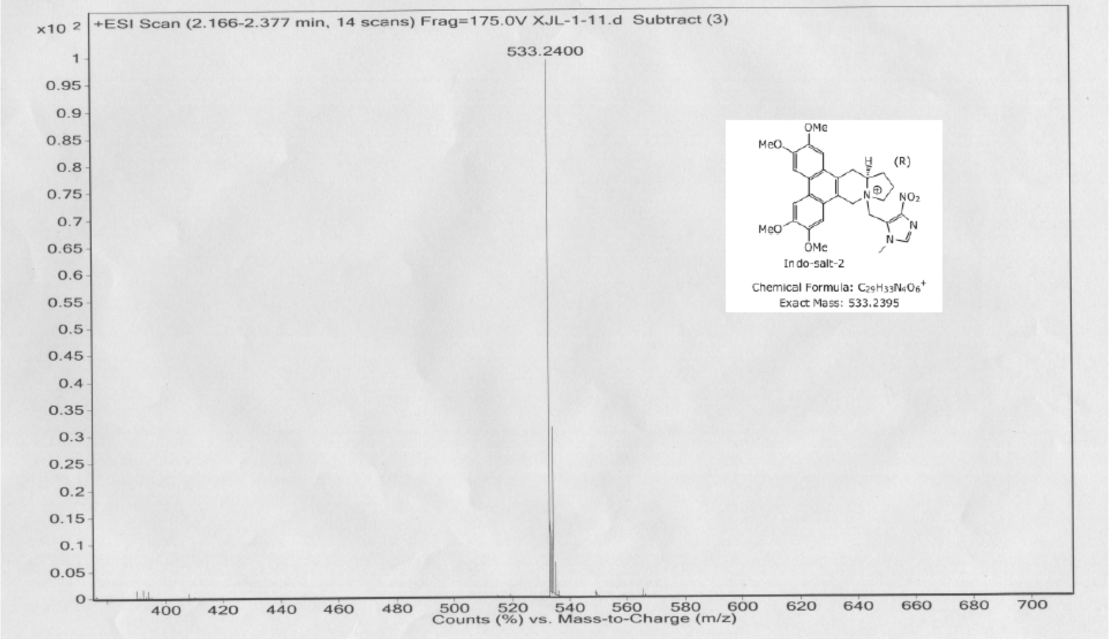

Supplement: Supplementary file 1 [file molecules-26-03327-s001.zip › molecules-1234279-supplementary.pdf]
